# Image Forgery Detection and Localization via a Reliability Fusion Map

**DOI:** 10.3390/s20226668

**Published:** 2020-11-21

**Authors:** Hongwei Yao, Ming Xu, Tong Qiao, Yiming Wu, Ning Zheng

**Affiliations:** 1 School of Cyberspace, Hangzhou Dianzi University, Hangzhou 310018, China; yaohomeway@gmail.com (H.Y.); mxu@hdu.edu.cn (M.X.); yimgwu@hotmail.com (Y.W.); nzheng@hdu.edu.cn (N.Z.); 2 Institute of Cyberspace Research, Zhejiang University, Hangzhou 310027, China

**Keywords:** digital image forensics, tampering detection and localization, convolution neural network (CNN), reliability fusion map (RFM)

## Abstract

Moving away from hand-crafted feature extraction, the use of data-driven convolution neural network (CNN)-based algorithms facilitates the realization of end-to-end automated forgery detection in multimedia forensics. On the basis of fingerprints acquired by images from different camera models, the goal of this paper is to design an effective detector capable of completing image forgery detection and localization. Specifically, relying on the designed constant high-pass filter, we first establish a well-performing CNN architecture to adaptively and automatically extract characteristics, and design a reliability fusion map (RFM) to improve localization resolution, and tamper detection accuracy. The extensive results from our empirical experiments demonstrate the effectiveness of our proposed RFM-based detector, and its better performance than other competing approaches.

## 1. Introduction

As digital and other communications technologies advance, digital images, videos and audio files can be conveniently acquired from various devices, ranging from the conventional closed-circuit television cameras (CCTVs), digital cameras to other Internet of Things (IoT) devices with image, video and audio capturing capabilities (e.g., Ring Doorbell Camera). Modifying an image has also become easier, due to the availability of inexpensive image, video and audio (collectively referred to as multimedia) editing software. Implications of forged multimedia files, for example using re-sampling [1,2] or copy-moving [3,4], include ownership infringement or fraudulent activities. For example, as recently as Sep 2019, “the CEO of an unnamed UK-based energy firm believed he was on the phone with his boss, the chief executive of the firm’s German parent company, when he followed the orders to immediately transfer €220,000 (approx. $243,000) to the bank account of a Hungarian supplier” (https://www.forbes.com/sites/jessedamiani/2019/09/03/a-voice-deepfake-was-used-to-scam-a-ceo-out-of-243000/). This necessitates the need to design an effective and robust forensic detector with the capability of providing reliable digital evidence.

The study of both source identification and tampering detection is a relatively mature topic [5,6,7] for details. Image tampering detection targets processing techniques, such as object removing or adding. Object forgery detection approaches can be divided into three classes: (i) splicing detection: given two images, one can detect if a region of a source image has been spliced into a target image [8,9,10,11,12,13]; (ii) copy-moving forgery detection: given an image, one can identify if an object is copied-and-pasted from one to another location [14,15,16,17]; and (iii) object removal detection: given an image, one can detect if an object of the source image has been removed [18,19,20].

There has been a recent trend of moving away from conventional hand-crafted feature extraction to using convolution neural network (CNN)-based extractors. However, some primitive CNN-based forensic detectors are generally not practical for a number of reasons, for example in terms of the robustness of feature extraction, and the resolution of tampering localization. Therefore, there have been efforts to design a pre-processing layer to enhance the robustness of feature extraction [21,22,23], and fusing multiple detectors based possibility maps [24] and single CNN-based reliability maps [25,26] to improve the resolution of tampering localization.

There still remain several limitations in the aforementioned approaches. First, most existing pixel-wise tampering detectors adopt an independent patch-based strategy rather than using the correlated information among patches. This results in insufficient statistical information required for feature extraction, especially on the edge of a forged region. In other words, we should emphasize on neighbor patches’ characteristics to facilitate the determination of the authenticity of an inquiry patch (a principle we consider in this work). Furthermore, the absence of statistical characteristics over flat areas (clear sky, blue ocean, etc.) results in estimation ambiguity, and results in degraded detection performance. In that case, the texture of the image content becomes a decisive factor for enhancing detection accuracy. Besides, with the rapid development of image-editing software, the remnants left by manipulation operation have a behavior similar to its pristine version (i.e., tampering traces are hard to detect). Therefore, how to reduce the probability of detection mismatch and improve the resolution of localization (controlled by the smallest unit of detection) remains an open problem.

To address that challenge, in this paper, we propose a novel end-to-end framework to improve the accuracy of tampering detection and localization, mainly for composite images edited from different imaging sources. The main idea behind the proposed method is that camera model-related artifacts can be successfully extracted from a typical image acquisition pipeline, leading to that our proposed reliability fusion map (RFM)-based detector can capture subtle manipulation traces (see Figure 9 for illustration). By designing a pre-processing module, together with a feature extraction module containing CNN module equipped with content-texture module, a feature vector with initial detection (Figure 10d) is effectively generated. More importantly, we design a reliability fusion map (RFM) to improve the localization resolution (Figure 10e). The effectiveness of our proposed method (The source code is available on Github: https://github.com/grasses/Tampering-Detection-and-Localization) is experimentally verified compared with the prior arts [23,26].

The remainder of this paper is organized as follows. Section 2 reviews the related literature. In Section 3, we describe our proposed framework, consisting of a pre-processing stage (high-pass filter), a feature extraction stage (CNN module equipped with content-texture module), and a reliability fusion stage (binary map RFM). Section 4 presents the numerical results over the benchmark dataset, and a comparative performance evaluation. Finally, Section 5 concludes this paper.

## 2. State of the Art

**Figure 1:**
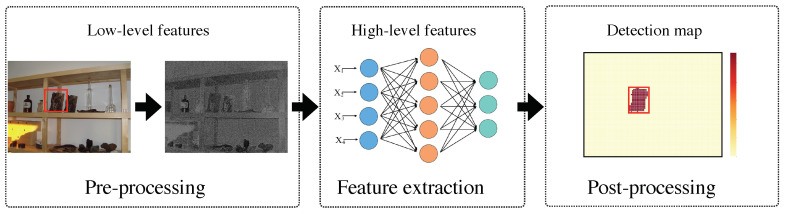
Generic framework for image tampering detection and localization, including image pre-processing, feature extraction, and post-processing.

A generic framework of tampering detection usually contains the following steps: pre-processing, feature extraction, and post-processing (see Figure 1). In general, low-level features are extracted in Stage 1; high-level features are extracted in Stage 2; Stage 3 plays a critical role in tampering detection and localization, that we mainly focus on in this paper. Next, let us generally review the relevant literature based on these three stages.

### 2.1. Pre-Processing Based Algorithms

Image pre-processing efforts have generally been put on how to manually design efficient constant convolution kernels, and meanwhile to train an effective feature extractor of capturing characteristics related to tampering traces. For instance, the research community has proposed constant filters to suppress the interference caused by edges and textures, and enhance the intrinsic features, such as using the median filter residual (MFR) [27], guided filtering for photo response non-uniformity noise (PRNU) [28], resampling detectors [29,30] and other forensic detectors based on steganalytic features like spatial rich model (SRM) [31]. It should be noted that the constant filter is good at accelerating convergence of a neural network, since the residual image obtained from a constant filter is content-independent.

Inspired by the aforementioned effective high-pass filter, some researchers utilized a pre-determined predictor to produce a series of residual pixels. Then, these residual pixels are exploited as low-level forensic features. High-level associations are formed by subsequent detection. For instance, Bayar and Stamm [22] combined a constant filter with a trainable convolutional filter in the pre-processing stage to enhance the robustness of detection. Subsequently, they used a new type of CNN layer (referred to as the constrained convolutional layer) for designing a universal detector [23]. Although this approach [23] reportedly achieved high detection accuracy, its theoretical performance for image tampering localization is still unknown. Moreover, each isolated patch-wise detection result is hardly analyzed together, leading to that the mismatched results of detection to some extent decrease the resolution of tampering localization (see Figure 9). However, in this paper, due to our proposed RFM algorithm, that limitation can be perfectly overcome.

### 2.2. Feature Extraction Based Algorithms

A number of feature extraction techniques have been proposed, such as those designed to distinguish camera fingerprints, leading to detection of camera model based tampered images. Ref. [32] proposed a CNN module to extract a noise residual, called noiseprint, which largely suppressed the scene content and enhanced camera model-related artifacts. Despite the promising results shown in [32], one has to keep in mind that the noiseprint can only be useful for camera model identification, but not for individual device identification. A large scale of feature extraction techniques leveraged other artifacts inherited in an image. By utilizing the information of chroma and saturation, Ref. [33] designed a Shallow Convolutional Neural Network (SCNN) to detect and localize the traces of low resolution tampered images. Ref. [34] investigated the features of manipulation especially artifacts near boundaries of manipulated regions. Then they proposed an encoder-decoder based network to exploit these traces. Some prior arts focused on designing the architecture of neural network to improve the manner of learning process and strengthen the effectiveness of feature extraction. Inspired by the mechanism of memory in human brain, Ref. [35] proposed a Ringed Residual U-Net (RRU-Net) to accelerate the convergence of the neural network. The RRU-Net was efficient in exploring the differences of image attributes between the pristine and tampered regions by using the contextual spatial information in an image. Ref. [36] proposed a densely connected CNN module to increase variations in the input of subsequent layers. The dense connectivity, which had better parameter efficiency than the traditional pattern, ensured the maximum information flow between layers in the network. Next, we will revisit some of the strategies proposed to improve resolution of tampering localization using high-level features.

### 2.3. Post-Processing Based Algorithms

In the stage of post-processing, one can utilize high-level features to obtain better localization resolution. The problem of tampering localization requires one to accurately specify forged region by minimizing the probability of patch-wise detection mismatch. In fact, tampering localization in a forged image is more difficult than merely binary classification between pristine and forged one.

Many prior works leveraged distinctive artifacts inherited in an image, for instance, based on sensor pattern noise [25,28,37], JPEG attributes [38,39], multiple techniques fusion [40,41,42,43]. Similar, the authors of [24] combined two existing forensic approaches (i.e., statistical feature-based and copy-moving forgery detectors) to obtain the tampering possibility map. Although such a method can deal with various manipulations, its usage in real-time scenario is limited due to its 18,157-dimensional high-level features.

CNN-based methods often employed one feature extractor coupled with confidence factors for detection. For instance, in [25], a two-tiered transfer learning-based approach was proposed for patch reliability estimation using camera model attribution, which achieved performance improvement in one single patch. However, the approach did not consider reliability of adjacent patches, and its theoretical performance on the whole image remains unknown. To mitigate the limitations, the authors in [26] used step-by-step clustering of camera-based CNN features. However, the localization resolution still needs to be improved. In addition, due to the extensive dependence of group constrained thresholds for filtering out nuisance noise, its robustness remains to be verified.

Existing approaches mainly focus on the generalized three stages in order to improve the performance of tampering detection and localization. During pre-processing stage, one accelerates the convergence of neural network and improves performance of feature extraction. In feature extraction stage, one utilizes an effective CNN to extract features characterizing tampering traces. In the post-processing stage, one reduces the mismatch result of detection, and improves the resolution of localization. It makes sense that different approaches have their unique advantages and limitations. Therefore, how to leverage the advantages of current arts for improving the accuracy of both detection and localization remains an ongoing challenge. In the following section, dependent of the powerful CNN, we will specifically present the design of an efficient RFM-based detector.

## 3. Proposed Method

**Figure 2:**
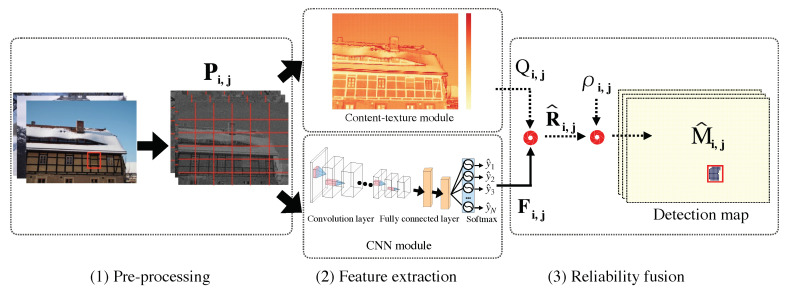
Flowchart of our proposed classifier.

The core idea behind our proposed method is that both tampering detection and localization are based on fingerprint discrimination among different camera models. Our proposed RFM-based detector is described below (see Figure 2): (i) pre-processing: we utilize a fixed high-pass filter to obtain full-size residual image, and then split the residual image into a set of 64×64 overlapped patches with stride of 32; (ii) feature extraction: we design the CNN module equipped with content-texture module, including each component for designing convolutional layer, fully-connected layer, and classification layer; (iii) reliability fusing: three significant factors are proposed to establish the binary map RFM for detecting tampered image and localizing forged region.

### 3.1. Pre-Processing

Let us assume that a pristine image is captured by an imaging device while its forged region is obtained from another. In order to remove interference from image content, a high-pass filter (see Equation (1)) formulated as:

(1) $$\begin{array}{c} F_{0}=\frac{1}{12}\left[\begin{array}{ccccc} -1 & 2 & -2 & 2 & -1\\ 2 & -6 & 8 & -6 & 2\\ -2 & 8 & -12 & 8 & -2\\ 2 & -6 & 8 & -6 & 2\\ -1 & 2 & -2 & 2 & -1 \end{array}\right] \end{array}$$

is used in the stage of pre-processing to extract a residual image of each inquiry image. We remark that the high-pass filter is efficient in accelerating convergence of neural network, and its performance has been verified in [44,45,46]. Subsequently, it is proposed to split the residual image I into 64×64 patches. All patches from a pristine image are captured by the same camera. On the contrary, patches from a forged image contain more than one fingerprint generated by different cameras. Then, we define $P_{i,j}$ as the extracted patch, and $i\in \{0,N_{1}-1\}$, $j\in \{0,N_{2}-1\}$, $N_{1}\times N_{2}$ denotes the total number of patches extracted from I (see Figure 2).

### 3.2. Feature Extraction

The establishment of the proposed feature extraction involves two main stages, namely the CNN module and the content-texture module where texture quality is designed to quantify the perceived texture of each patch. In fact, it is worth noting that our proposed CNN module deals with the patch as the smallest calculation unit.

#### 3.2.1. CNN Module

**Figure 3:**
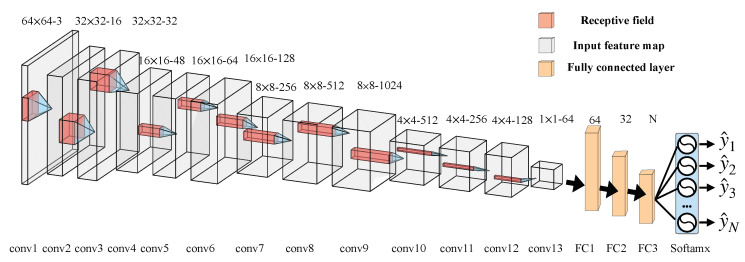
Architecture of the convolution neural network (CNN) module, with 13 conventional layers, three fully-connected layers and a softmax layer.

A typical CNN module consists of stacked convolutional layers, and fully connected layers, followed by a softmax classifier (or classification layer) (see Figure 3 and Table 1 for details). The stacked convolutional layers can be defined as follows:

(2) $$f^{n}\left(P_{i,j}\right)=f_{\mathrm{pooling}}\left(f_{\mathrm{activation}}\left(f^{n-1}\left(P_{i,j}\right)*w^{n}+b^{n}\right)\right),$$

where a patch $P_{i,j}$ is fed into our CNN module, “*” means the convolution operation, $f^{n}\left(\cdot \right)$ denotes an output of the $n_{th}$ convolutional layer, and $w^{n}$ and $b^{n}$ are shared weights and bias parameter. $f_{\mathrm{pooling}}\left(\cdot \right)$ represents a pooling layer, which controls the representation dimension by reducing the amount of parameters and computation in the CNN module. It avoids the problem of overfitting. $f_{\mathrm{activation}}\left(\cdot \right)$ represents an activation function, aiming at activating effective units while suppressing invalid units.

Next, fully connected layers featured by the network parameters play an important role in the establishment of classification layer. The fully connected layer feeds the features, that are extracted from the convolutional layer, back to a typical softmax classifier. It is worth noting that each output of the node from the softmax classifier is a probability, serving as the discriminative factor for our classification. In the stage of backpropagation, the cross-entropy error function (namely loss funtion) is used to measure the distance between probability for each classification and original distribution, which can be defined as follows:

(3) $$\underset{\Theta }{\mathrm{argmin}}\;L\left(y,\hat{y};\Theta \right)=-\sum_{i}^{N}y_{i}\times \log\left({\hat{y}}_{i}\right),$$

where ${\hat{y}}_{i}$ denotes the probability for *i*-th classification; Θ represents the parameters of neural network. By minimizing the objective function L, the parameters of neural network is refined with Stochastic Gradient Descent (SGD) automatically. It should be noted that the goal of loss function in this paper is to discriminate among different camera models.

In this paper, we adopt the CNN architecture similar to our prior work [47]. Since the input data, referring to as patches, are not very large, the neural network should be good at analyzing difference between the pixel and its neighboring counterparts, and have a strong predictive ability to characterize feature maps. In general, a too-wide network architecture cannot fully learn the feature map; a too-deep network architecture might cause increment of the computational complexity. Hence, our proposed network is neither too deep nor too wide. In this context, we mainly focus on the design of fusion map for splicing detection and localization, but not for specific description of CNN module (the readers may refer to [47] for details).

Different our previous work [47] mainly analyzing the image features characterizing different source camera models, in this paper, we adopt a CNN architecture equipped with content-texture module, and leverage a reliability fusion map to refine extracted features for dealing with the problem of tampering detection and localization.

#### 3.2.2. Content-Texture Module

When dealing with a low texture patch, the performance of the CNN module should be further enhanced. Inspired by the algorithm proposed in [37], we use the texture quality measure standard to define a patch texture, formulated as follows:

(4) $$Q=\frac{1}{3}\sum_{c\in R,G,B}^{}\left[\alpha \times \beta \left(\mu_{c}-{\mu_{c}}^{2}\right)+\left(1-\alpha \right)\left(1-e^{\gamma \sigma_{c}}\right)\right],$$

where three parameters α, β and γ are used to assign the weights into $\mu_{c}-\mu_{c}^{2}$ and $1-e^{\gamma \sigma_{c}}$. $\mu_{c}$ and $\sigma_{c}$, c∈{R,G,B} respectively denote the mean and standard deviation of $P_{i,j}$ for each color channel. In our experiment, α=0.7, β=4 and γ=ln(0.01). $Q_{i,j}$ for each patch is normalized into the range $\left[0,1\right]$. As a decisive factor, texture quality suppresses ambiguous classification of CNN over the low-texture regions while further enhancing prediction accuracy in high-texture regions, leading to decreasing the mismatch of classifications.

### 3.3. Reliability Fusing

**Figure 4:**
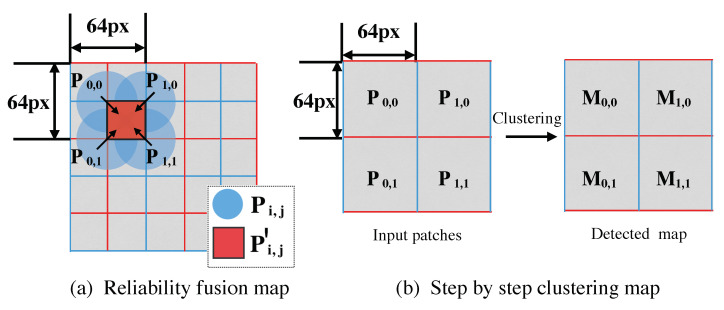
Illustration of the reliability fusion map (RFM) algorithm pipeline (**a**), and step by step clustering approach of [26] (**b**). “px” is the abbreviation of “pixel”.

One cannot guarantee that all regions contain adequate statistical information for tampering localization, especially dealing with low-texture regions. In addition, the output result from our CNN module contains the probability vector for each camera model, meaning that it is more than just a binary (true or false) classification. The detection result of the adjacent patches may influence that of the central inspected patch. For instance, if the result of the patch generated by the CNN module has the large probability as a tampering sample while the results of its adjacent neighbors as pristine, it is reasonable that the probability of detection mismatch has increased. To achieve improvement in detection and localization accuracy, the reliability-fusing operation is thus proposed in this context. For clarity, we illustrate an example of the proposed RFM algorithm (see Figure 4). Let us give the specific description of RFM algorithm, involving three following factors:

- Patch texture $Q_{i,j}$. The parameter $Q_{i,j}$ can provide information about content texture of inquiry patch, which tends to be low for flat patches and high for patches with high variance. Since CNN module cannot perform in low-texture regions as well as in high-texture regions, let us accordingly decrease CNN confidence $F_{i,j}$ in low-texture regions.
- CNN confidence $F_{i,j}$. $F_{i,j}$ represents the output result of the CNN module extracted from $P_{i,j}$, among which sum of all vectors equals to 1. Rather than truncating confidence $F_{i,j}$ by an empirical threshold, our proposed algorithm combines the CNN confidence for each patch, meaning that the algorithm accumulates the CNN confidence $F_{i,j}$ of adjacent patches around the inspected (or central) patch.
- Density distribution $\rho_{i,j}$. $\rho_{i,j}$ represents a tampering ratio of *K* adjacent patches. $\rho_{i,j}$ is proposed to remove the mismatched results generated by the CNN confidence $F_{i,j}$. The larger $\rho_{i,j}$ indicates the more forged adjacent patches around the inspected patch.

Next, we will extend the specific reliability fusing procedure (RFM algorithm) to obtain the binary map RFM.

#### 3.3.1. Fusing $Q_{i,j}$ and $F_{i,j}$

Relying on $Q_{i,j}$, overlapped adjacent patches, referring to ($P_{0,0}$, $P_{0,1}$, $P_{1,0}$, and $P_{1,1}$), jointly re-identify the central patch. Therefore, half of detection unit size with 32×32 is reduced (see Figure 4a), compared with the general clustering algorithm with 64×64 (see Figure 4b). Then, the formula is defined as follows:

(5) $${\hat{R}}_{i,j}=\sum_{a=0}^{1}\sum_{b=0}^{1}\left(\frac{Q_{i+a,j+b}}{\sum \sum Q}\times F_{i+a,j+b}\right),$$

where $F_{i+a,j+b}$ represents the CNN confidence, and $Q_{i+a,j+b}$ is the adjacent patch texture. ${\hat{R}}_{i,j}$ denotes the reliability vector of the fused central patch ${P^{{}^{\prime }}}_{i,j}$, which is a re-estimation of the CNN confidence for four adjacent patches (see Figure 4a), relying on the assigned weights generated by *Q*. The reason why we choose four adjacent neighbors rather than only one used in existing methods such as [26] is twofold: (1) if only one nearest neighbor is considered, the localization accuracy may potentially decrease caused by incorrect classification; (2) The utilization of four adjacent neighbors effectively improves the localization resolution.

#### 3.3.2. Fusing ${\hat{R}}_{i,j}$ and $\rho_{i,j}$

We convert the reliability vector ${\hat{R}}_{i,j}$ into a tampering binary mask ${\hat{M}}_{i,j}\in \left\{0,1\right\}$, based on the majority voting of the reliability vectors generated by neighboring patches. When ${\hat{M}}_{i,j}=0$, ${P^{{}^{\prime }}}_{i,j}$ is pristine; on the contrary, when ${\hat{M}}_{i,j}=1$, ${P^{{}^{\prime }}}_{i,j}$ is forged. Next, $\rho_{i,j}$ can be calculated using the following equation:

(6) $$\rho_{i,j}=\frac{\sum \hat{M}}{K},$$

where *K* is the number of adjacent patches for ${P^{{}^{\prime }}}_{i,j}$, and we set *K* as 8 to facilitate detection in practice. If $\rho_{i,j}$ is smaller than $\tau_{1}$, it is proposed to refine detected region in the mask by setting all inspected patches as pristine, which can be formulated as follows:

(7) $${\hat{M}}_{i,j}=0\;\mathrm{if}\;\rho_{i,j}<\tau_{1},\;$$

where $\tau_{1}\in \left[0,1\right]$ denotes a threshold. Note that when $\tau_{1}=0$, we do not take $\rho_{i,j}$ into consideration; when $\tau_{1}=1$, the inspected patch requires *K* forged adjacent patches. Then, we can generate the binary map RFM through ${\hat{M}}_{i,j}$. For clarity, the visualization result of RFM is illustrated in Figure 9.

#### 3.3.3. Designing Binary Classifier

To automatically realize the end-to-end detection, we introduce $\tau_{2}$ to determine whether image I is forged or not by counting the number of forged patches:

$$\left\{\begin{array}{cc} I\;\mathrm{is}\;\mathrm{pristine} & \;\mathrm{if}\;\mu_{\hat{M}}\leq \tau_{2}\\ I\;\mathrm{is}\;\mathrm{forged} & \;\mathrm{if}\;\mu_{\hat{M}}>\tau_{2} \end{array}\right.$$

where a threshold $\tau_{2}\in \left[0,1\right]$ controls the number of forged patches in an inquiry image. $\mu_{\hat{M}}$ denotes the averaged tampering rate of image I, which is calculated using the below equation:

(8) $$\mu_{\hat{M}}=\frac{\sum \sum {\hat{M}}_{i,j}}{N_{1}\times N_{2}},$$

where $N_{1}\times N_{2}$ denotes the total number of patches extracted from I.

## 4. Experimental Results

In order to comprehensively evaluate the performance of our proposed RFM-based detector, we focus on pre-processing effectiveness, binary tampering detection, and forgery localization. The results are compared with the competing state-of-the-art approaches. First, we will describe the database used in our evaluation.

We utilize the benchmark Dresden Database [48], which consists of more than 16,000 images from 26 different camera models depicting a total of 83 scenes. In our evaluation, we randomly selected 18 camera models from the Dresden Database, and split them into a training set $D_{T}$, a validation set $D_{V}$ and an evaluation set $D_{E}$.

Images both from dataset $D_{T}$ and $D_{V}$ were first divided into 64×64 overlapped patches. Then, we trained the CNN module in Section 3.2 in virtue of the Stochastic Gradient Descent [49]. We randomly selected 2700 images (150 images per model) as the training set $D_{T}$, and another 1800 images (100 images per model) as the validation set $D_{V}$. Meanwhile, we modified 500 images using the cross-model strategy from $D_{V}$, and randomly chose another 500 images from $D_{V}$ as pristine samples, with a total of 1000 images (over 2,000,000 patches) as the evaluation set $D_{E}$. In the following, we will describe the cross-model strategy.

The procedure of generating forged images is described in Algorithm 1. We first randomly select 500 images from nine camera models as group *A*, and 500 images from the remaining camera models as group *B*. Subsequently, we will select an image $I_{tmp}$ from group *B* to tamper a host image $I_{rev}$ from group *A*. The next step is to generate a blank mask M with the same size of $I_{rev}$. Then, we crop a random rectangle region Q with the size of w×h ($w\in \left[128,1024\right]$ and $h\in \left[128,1024\right]$) from $I_{tmp}$, and splice it into a random location of $I_{rev}$ as forged image $I_{forge}$. Finally, we update M to mark the tampering region, and respectively, save $I_{forge}$ and M as forged image and ground truth mask.

Finally, it is proposed to validate our algorithm based on the trained CNN module. It should be noted that we use the same forged dataset in our experiments for fair comparison. We implement the experiments on a single Nvidia GPU card of type GeForce GTX 1070, with its built-in Deep Learning Tensorflow.
**Algorithm 1:** Procedure of generating forged images
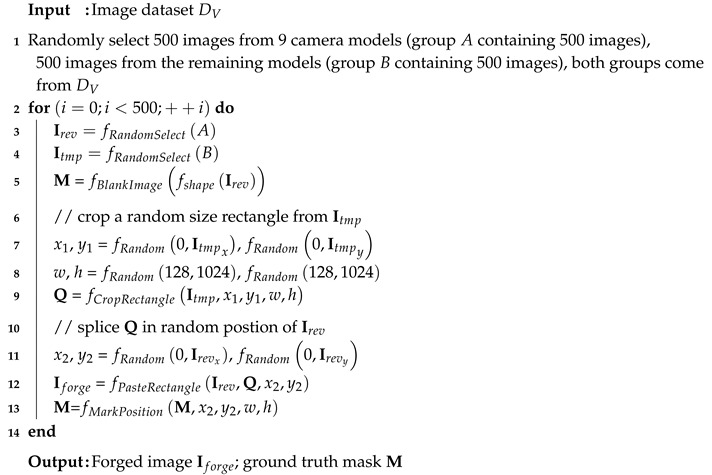


### 4.1. Pre-Processing Performance Evaluation

In the first evaluation, we intend to understand the knowledge hidden in the pre-processing stage. We experimentally compare our proposed high-pass filter (RFM-CNN for abbreviation), trainable pre-processing filter (Constrained-CNN) [23], and our previous work (SCI-CNN) [47] without pre-processing operation, to validate the effectiveness of pre-processing performance. It should be noted that RFM-CNN represents the key step of our proposed RFM-based detector, which only contains pre-processing and feature extraction stages. To this end, they were first trained with $D_{T}$ and then evaluated by $D_{E}$.

**Figure 5:**
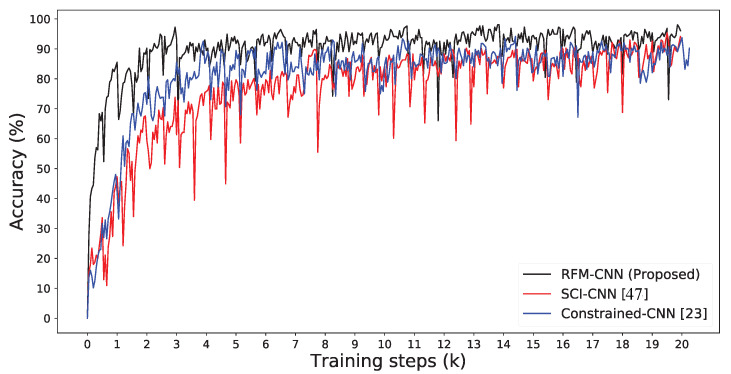
Accuracy curves on training dataset ($D_{T}$) for Constrained-CNN [23], SCI-CNN [47] and RFM-CNN proposed in this work.

Figure 5 depicts the training accuracy curves for our proposed RFM-CNN, SCI-CNN [47] and Constrained-CNN [23].

For fair comparison, the same CNN architecture is adopted in this experiment. It should be noted that the accuracy here is used for evaluating the classification performance of images from various camera models (i.e., patch-wise accuracy), different from the definition of accuracy of tampering localization in the following subsection. We observe that RFM-CNN had an average accuracy of over 90% using only about 3000 training steps, which achieved faster convergence than Constrained-CNN and SCI-CNN. Due to the constant pre-processing filter, the RFM-CNN framework was able to leverage the CNN to extract inherent characteristics of an image. Besides, it implies that the better-performed classification for identifying camera model undoubtedly leads to higher accuracy of tampering detection and localization.

**Figure 6:**
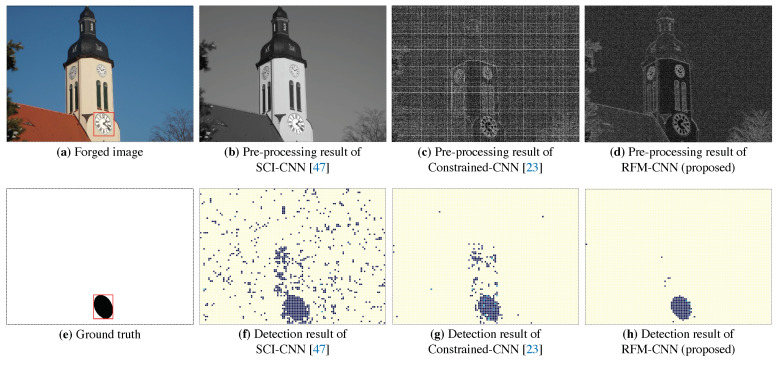
Tampering localization with different pre-processing stages: (**a**) forged image; (**e**) ground truth; (**b**) SCI-CNN denoting grayscale input image without pre-processing operation; (**c**) Constrained-CNN; (**d**) RFM-CNN with pre-processing operation; (**f**–**h**) visualization results generated by different methods.

As Figure 6 reports, we illustrate the detection visualization results between the proposed RFM-CNN and the other pre-processing strategies. We inserted a red bounding box labeling the tampering region. It should be noted that the pre-processing result of SCI-CNN is actually grayscale version of inspected color image, since the pre-processing operation was not adopted in that method. One can also observe that both RFM-CNN and constrained-CNN were capable of suppressing low-frequency content while enhancing high-frequency content. Moreover, according to magnitude of mismatch detection, RFM-CNN had a higher ability of feature extraction using constant filter, compared with Constrained-CNN and SCI-CNN. Therefore, from Figure 5 and Figure 6, one can conclude that the proposed RFM-CNN performs effectively in accelerating the convergence of neural network and assisting the CNN module to better extract features precisely.

Next, we analyze the importance of adopting the pre-processing stage prior to CNN. When extracting intrinsic features, it is required to suppress content-related features. Thus, it is proposed to enhance the effectiveness of the CNN equipped with pre-processing stage for capturing image intrinsic fingerprints. Moreover, an efficient pre-processing operation, referring to as an effective high-pass filter, can further improve the convergence and efficiency in feature extraction of CNN. For instance, an appropriate constrained filter has verified its effectiveness of improving detection performance (see [23]).

### 4.2. Tampering Detection

**Figure 7:**
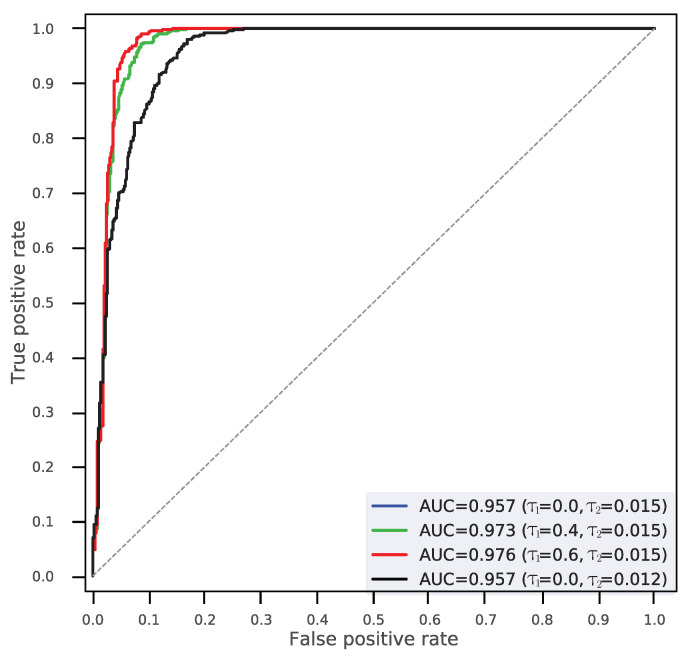
ROC curves of tampering detection results using our RFM-based detector with various thresholds $\tau_{1}$ and $\tau_{2}$.

In this section, we presented the performance evaluation of the RFM algorithm on tampering detection. The proposed CNN was first trained using $D_{T}$ and then tested with $D_{E}$. We adjusted thresholds $\tau_{1}$ and $\tau_{2}$ to obtain different results. Table 2 illustrates the detection accuracy (ACC), true positive rate (TPR) and false positive rate (FPR) of the RFM-based detector. Figure 7 describes the ROC curves under different $\tau_{1}$ and $\tau_{2}$. In this experiment, the ACC denotes tampering detection accuracy (i.e., binary classification) for proposed RFM method, which can be formulated as follows:

(9) $$ACC=\frac{TP+TN}{N},$$

where TP denotes true positive and TN denotes true negative, *N* denotes the total number of images in $D_{E}$. Besides, TPR can be formulated as follows:

(10) $$TPR=\frac{TP}{TP+FN}.$$

It should be noted that $\tau_{1}$ plays an important role in reducing mis-classified patches. Additionally, $\tau_{2}$ plays a critical role in determining the number of detected patches for identifying a forged image.

Table 2 describes the performance of our proposed RFM-based detector (i.e., an average ACC of 92.2%). As Table 2 illustrates, when $\tau_{1}$ decreased from 0.6 to 0, the ACC decreased from 94.9% to 90.4%. In other words, the RFM in the fusing stage can effectively reduce mis-classification, and meanwhile refine tampering detection. Figure 7 describes the ROC curves obtained from different threshold $\tau_{1}$ and $\tau_{2}$, where TPR achieves high values even at a very low FPR. Thus, the findings supported the fact that our detector can precisely identify forged images with a low mis-classification rate.

**Figure 8:**
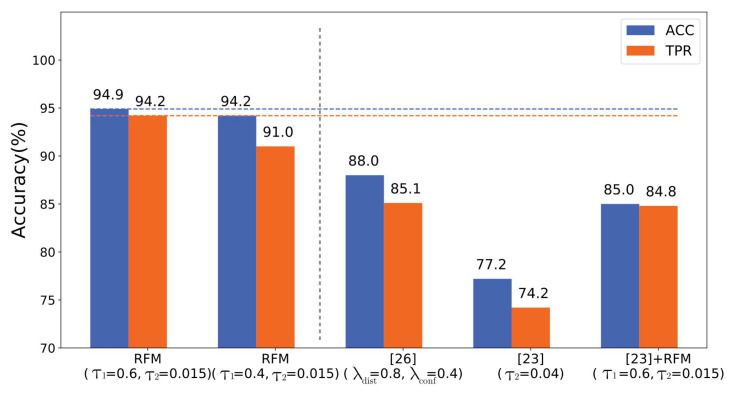
Accuracy (ACC), true positive rate (TPR) of proposed method with various thresholds $\tau_{1}$, $\tau_{2}$ (on the left of the gray dashed line) and the other competing algorithms (on the right of the gray dashed line). Blue and orange dashed lines denote the best ACC and TPR results of our proposed RFM-based detector, respectively.

Moreover, we compared the proposed RFM-based detector with [26] and [23], where [26] focused on clustering CNN features and [23] had a trainable pre-processing filter (Constrained CNN). For a fair comparison, the same pre-trained CNN module was applied to our proposed method and the approach of [26]. Meanwhile, we added an additional experiment by adopting the RFM algorithm followed by the CNN output of [23] (see [23]+RFM in Figure 8). We used both ACC and TPR as the evaluation metrics to complete the comparison experiments.

Figure 8 presents the detection results of the RFM-based detector with various thresholds $\tau_{1}$ and $\tau_{2}$, together with the other prior-art methods. Compared with methods proposed in [26] and [23], the RFM-based detector achieved the best accuracy of 94.9% when $\tau_{1}=0.6$. Additionally, when we adopted the RFM algorithm to refine CNN features of [23], both ACC and FPR gained a remarkable enhancement. The main reason is that [23] adopts the strategy based on each isolated patch without taking features of adjacent patches into consideration, while our proposed RFM algorithm reduces the mis-classified result caused by one single patch, and meanwhile improves the accuracy.

### 4.3. Tampering Localization

We then compared the performance of our RFM-based detector with [26] for tampering localization. The CNN module was trained with the set $D_{T}$, and then verified using $D_{E}$. For the evaluation metrics, we used both local and global detection accuracy. The local accuracy refers to the ratio of the number of detected forgery patches to that of all the forgery patches; the global accuracy refers to the ratio of the number of correctly-classified patches (both forgery and pristine patches) to that of all the patches (a full-size image). It is worth noting that the local accuracy only depends on tampering region, and serves as an evaluation metric to evaluate localization resolution. The global accuracy plays a critical role in evaluating the patch-wise detection performance.

**Figure 9:**
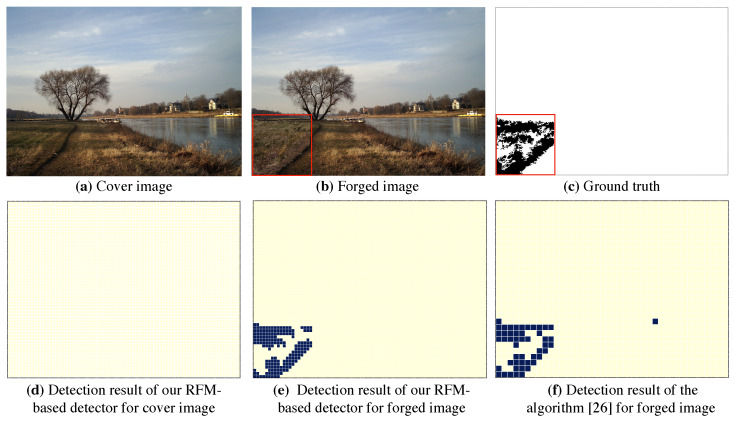
Comparison of localization performance between our RFM-based detector and the algorithm of [26].

Table 3 reports the results of tampering localization. It is observed that our RFM-based detector outperforms that of [26], with an average accuracy of over 90% (local accuracy), better than around 70% from [26]. That is, our proposed algorithm achieved significant improvement in the resolution of localization. Meanwhile, as Figure 9 illustrates the visualization results, the RFM-based detector had a higher resolution of localization, namely effective in locating the subtle tampering region. While our proposed RFM-based detector cannot perform as well as that of [26] in global accuracy. Nevertheless, Table 3 and Figure 9 empirically verify that our proposed RFM-based detector performs better in the resolution of localization.

**Figure 10:**
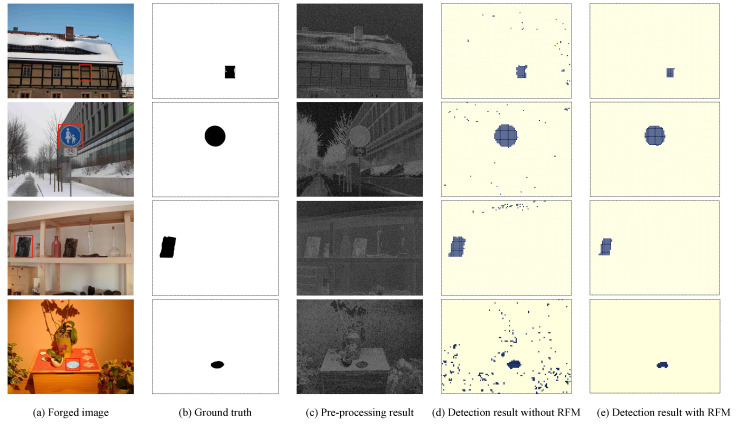
Tampering localization using our proposed RFM-based detector; from left to right: (**a**) forged image, (**b**) ground truth, (**c**) pre-processing result, (**d**) detection result without RFM (only relying on feature extraction), and (**e**) detection result with RFM (by adopting post-processing procedure).

A better insight on the result of each step can be demonstrated by a visual inspection of the examples of Figure 10. When only relying on the extracted features from CNN, one can observe that a large-scale mismatched patches labeled as dispersive colorized rectangles are scatted on the binary map (see Figure 10d). By adopting our proposed RFM algorithm, those mismatched patches can be filtered and refined (see Figure 10e), leading to more accurate tampering localization. It should also be noted that the tampering traces of examples in Figure 10 are hardly visually noticeable, which further highlights the powerful superiority of our proposed RFM-based detector.

## 5. Conclusions

The resolution of forgery localization is becoming more challenging for digital image forensics. Thus, in this paper, relying on CNN, we presented an RFM-based detector for authenticating a forged image and localizing tampering region. Specifically, in order to improve the accuracy of both tampering detection and localization resolution, we focused on the design of high-pass filter, the establishment of CNN architecture, and the construction of reliability fusion map, which mainly relies on patch texture, CNN confidence, and density distribution. Extensive evaluation results empirically demonstrated that our proposed RFM-based detector outperforms the prior arts in the resolution of localization.

However, the tampering technique is also advancing with the rapid development of image-editing software. Therefore, it is required to design an updated forensic detector for addressing the new challenge. Recently, a bunch of high-efficient detectors equipped with the new algorithms have been proposed to improve the performance of tampering detection and localization [50,51,52]. In our future work, we intend to further investigate the feature extractor characterizing the camera instance (not only focusing on the camera model) for widely tampering detection.

## Figures and Tables

**Table 1:** Configuration of each convolutional layer in Figure 3.

ID	Input Size	Configuration	Type
conv 1	64 × 64-3	stride = 2, ksize = 8 × 8	conv+ReLU
conv 2	32 × 32-16	stride = 1, ksize = 8 × 8	conv+ReLU
conv 3	32 × 32-32	stride = 2, ksize = 6 × 6	conv+ReLU
conv 4	16 × 16-48	stride = 1, ksize = 6 × 6	conv+ReLU+maxpool
conv 5	16 × 16-64	stride = 1, ksize = 3 × 3	conv+ReLU
conv 6	16 × 16-128	stride = 2, ksize = 3 × 3	conv+ReLU
conv 7	8 × 8-256	stride = 1, ksize = 3 × 3	conv+ReLU+maxpool
conv 8	8 × 8-512	stride = 2, ksize = 3 × 3	conv+ReLU
conv 9	8 × 8-1024	stride = 2, ksize = 3 × 3	conv+ReLU+maxpool
conv 10	4 × 4-512	stride = 1, ksize = 1 × 1	conv+ReLU
conv 11	4 × 4-256	stride = 1, ksize = 1 × 1	conv+ReLU
conv 12	4 × 4-128	stride = 2, ksize = 1 × 1	conv+ReLU
conv 13	1 × 1-64	stride = 2, ksize = 1 × 1	conv+ReLU+maxpool

**Table 2:** Results of tampering detection with various thresholds $\tau_{1}$ and $\tau_{2}.$

Threshold	ACC	TPR	FPR
$\tau_{1}$ = 0.0 $\tau_{2}$ = 0.015	0.904	0.828	0.020
$\tau_{1}$ = 0.4 $\tau_{2}$ = 0.015	0.942	0.910	0.026
$\tau_{1}$ = 0.6 $\tau_{2}$ = 0.015	**0.949**	**0.942**	0.044
$\tau_{1}$ = 0.6 $\tau_{2}$ = 0.012	0.892	0.792	**0.008**
Average	0.922	0.868	0.025

**Table 3:** Tampering localization comparison between our RFM-based detector and the algorithm of [26].

Method	Threshold	Local Accuracy	Global Accuracy	Resolution
RFM-based	$\tau_{1}$ = 0.4	**0.905**	0.954	32×32
RFM-based	$\tau_{1}$ = 0.6	**0.907**	0.955	32×32
[26]	$\lambda_{\mathrm{dist}}$ = 0.7, $\lambda_{\mathrm{conf}}$ = 0.2	0.712	**0.982**	64×64
[26]	$\lambda_{\mathrm{dist}}$ = 0.7, $\lambda_{\mathrm{conf}}$ = 0.0	0.734	**0.983**	64×64
